# Supplementary material for: Nutrient and Chemical Contaminant Levels in Five Marine Fish Species from Angola—The EAF-Nansen Programme
Source: Foods. 2020 May 14;9(5):629. doi: 10.3390/foods9050629 (PMC7278876; doi:10.3390/foods9050629)
Supplement: Supplementary file 1 [file foods-09-00629-s001.zip › Table S1.docx]

Supplementary materials

**Table S1**: Analytical values for selected nutrients and contaminants in composite samples (n=5) of fillets of *Lagocephalus laevigatus* (smooth puffer) from Angola (mean ± standard deviation)

|  |  | **Unit** | ***Lagocephalus laevigatus*** |
| --- | --- | --- | --- |
| Proximate composition | Moisture | % | 73.2 ± 0.3 |
|  | Protein | g/100 g | 23 ± 0.0 |
|  | Total fat | g/100 g | 2.2 ± 0.3 |
| Minerals | Calcium (Ca) | mg/100 g | 3.7 ± 0.1 |
|  | Iron (Fe) | mg/100 g | 0.23 ± 0.01 |
|  | Iodine (I) | mg/100 g | 13.7 ± 3.0 |
|  | Potassium (K) | mg/100 g | 596 ± 15 |
|  | Magnesium (Mg) | mg/100 g | 30.8 ± 0.8 |
|  | Sodium (Na) | mg/100 g | 51 ± 1.1 |
|  | Phosphorus (P) | mg/100 g | 324 ± 8.9 |
|  | Selenium (Se) | mg/100 g | 20.6 ± 1.1 |
|  | Zinc (Zn) | mg/100 g | 0.60 ± 0.06 |
| Contaminants | Arsenic (As) | mg/kg | 1.82 ± 0.30 |
|  | Cadmium (Cd) | mg/kg | 0.002 ± 0.001 |
|  | Mercury (Hg) | mg/kg | 0.074 ± 0.027 |
|  | Lead (Pb) | mg/kg | 0.005 ± 0.000 |
